# Dexmedetomidine versus midazolam on cough and recovery quality after partial and total laryngectomy – a randomized controlled trial

**DOI:** 10.1186/s12871-020-01168-7

**Published:** 2020-09-28

**Authors:** Rui Xu, Yun Zhu, Yi Lu, Wenxian Li, Jie Jia

**Affiliations:** 1 Department of Anesthesiology, The Eye, Ear, Nose and Throat Hospital of Fudan University, Shanghai Medical College of Fudan University, Fenyang Road #83, Shanghai, 200031 People’s Republic of China; 2 Department of Oro-maxillofacial Head and Neck Oncology, Shanghai Ninth People’s Hospital, Shanghai Jiao Tong University School of Medicine, Shanghai Key Laboratory of Stomatology, Shanghai, China

**Keywords:** Dexmedetomidine, Midazolam, Recovery quality, Laryngectomy

## Abstract

**Background:**

During emergence from anesthesia after partial and total laryngectomy, excessive airway reflex and systemic hypertension may lead to subcutaneous emphysema, hemorrhage or pneumothorax.

**Methods:**

American Society of Anesthesiologist physical status III and IV male adults undergoing elective laryngectomy were recruited and randomly allocated to receive either dexmedetomidine (group D) or midazolam (group M). The primary outcome was incidence and severity of cough. Pulse oximetry results (SpO_2_), heart rate (HR), systolic blood pressure (SBP), and diastolic blood pressure (DBP) were also recorded. The visual analog scale and the Ramsay sedation scale were recorded at the points of wakefulness and departure from the post-anesthesia care unit (PACU). Rescue analgesia consumption, the time of spontaneous breath recovery, duration of the PACU stay, and the incidence of adverse effects were also recorded.

**Results:**

The prevalence of no coughing was significantly higher in group D than in group M at the points of wakefulness and departure. HR, SBP, and DBP were significantly lower in group D compared with group M, and SpO_2_ was significantly higher in group D than in group M at the moment of laryngectomy. Pain scores were lower in group D than in group M. The Ramsay score at the point of wakefulness was higher in group D than in group M. There was no difference in time to spontaneous breathing recovery, duration of the PACU stay, and incidence of adverse effects.

**Conclusions:**

Compared with midazolam, dexmedetomidine is an effective alternative to attenuate coughing and hemodynamic changes with a low incidence of adverse events during emergence from anesthesia after partial and total laryngectomy.

**Trial registration:**

NCT03918889, registered at clinicaltrials.gov, date of registration: March 28, 2019.

## Background

Laryngeal carcinoma is one of the most common malignant tumors worldwide and usually requires head and neck surgery [1, 2]. A study by the International Agency for Research on Cancer reported 177,422 new laryngeal cancer cases and 74,771 cancer-related deaths in 2018 [3]. The treatment of laryngeal carcinoma has largely improved in recent years [4]. Partial and total laryngectomy is considered to be the most effective method, except for early-stage laryngeal carcinoma.

After surgery, air no longer passes through the upper respiratory tract, and without warming, humidifying, and filtering, air directly causes irritation of the trachea-bronchial mucosa. A tracheostomy tube is also a strong stimulus to the tracheal mucosa. Coughing can lead to subcutaneous emphysema, pneumothorax, surgical bleeding, and lung intercostal hernia [5]. Therefore, minimal coughing and smooth emergence should be achieved.

Dexmedetomidine is a highly selective α_2_ adrenoceptor agonist. Several studies have reported that dexmedetomidine may improve sympathetic tone, sedation, and analgesia without respiratory inhibition [6, 7]. To the best of our knowledge, there has been no study comparing recovery profiles between dexmedetomidine and midazolam after partial and total laryngectomy. Although the main drugs studied in our research are not commonly utilized during anesthesia for head and neck procedures, patients with tracheotomy after laryngeal carcinoma operation were chosen because of the strong discomfort and restlessness caused by the tracheotomy cannula, which is, however, guaranteed to keep the airway completely open. The aim of our study was to compare the effects of dexmedetomidine and midazolam on hemodynamics and recovery after partial and total laryngectomy.

## Methods

### Study design

**Fig. 1:**
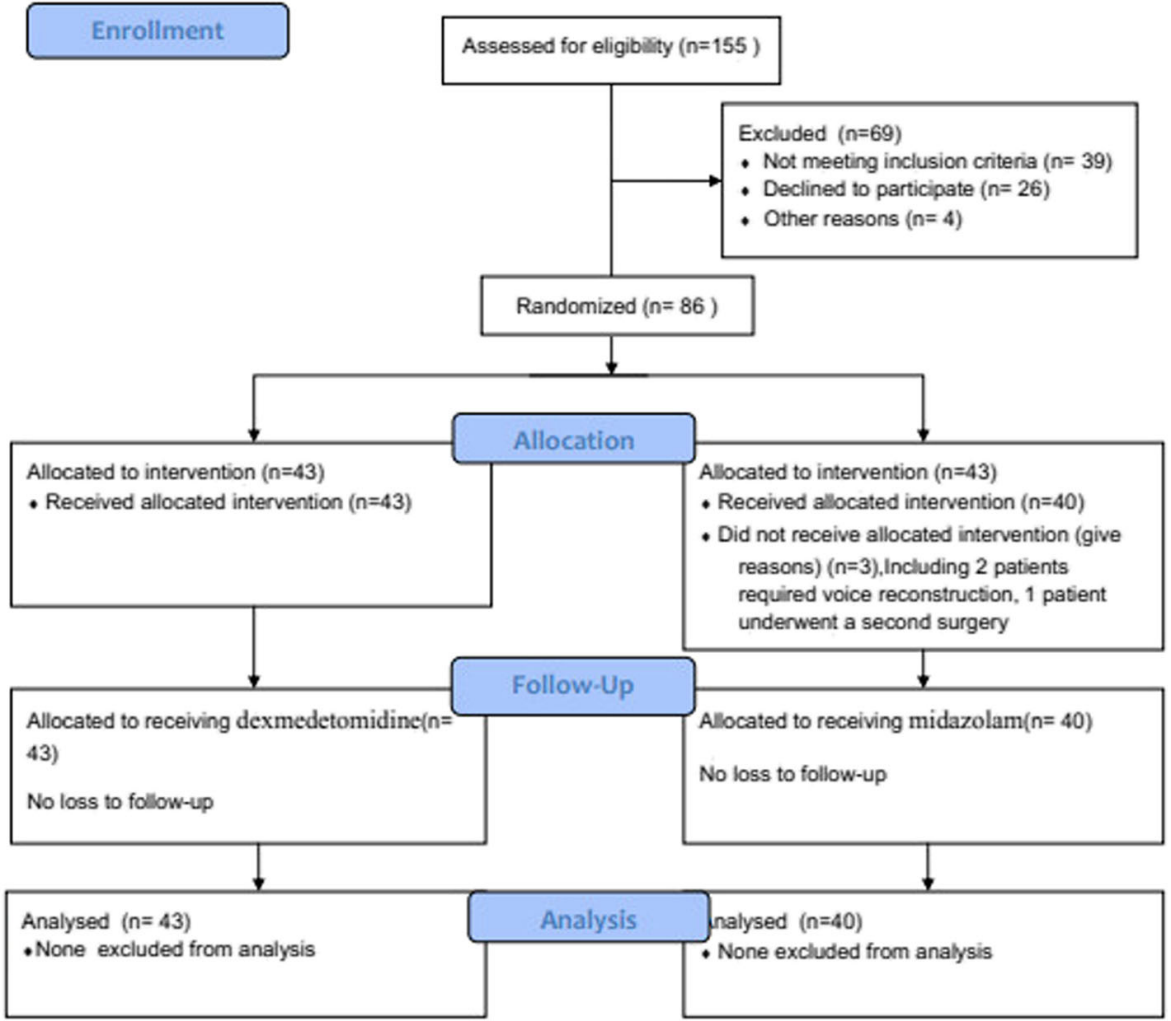
Consort flow diagram

This study was approved by the Ethics Committee of the Eye, Ear, Nose, and Throat Hospital of Fudan University, Shanghai, China (2013005). Written informed consent was obtained from all subjects participating in the trial. This prospective, randomized, double-blind, single-center clinical trial was registered prior to patient enrollment at clinicaltrials.gov (NCT03918889, principal investigator: Rui Xu, date of registration: March 28, 2019) and performed at the Department of Anesthesiology in the Eye, Ear, Nose, and Throat Hospital of Fudan University. All procedures adhered to the applicable CONSORT guidelines (Fig. 1).

Patients were randomly allocated to either the dexmedetomidine group (group D) (*n* = 43) or the midazolam group (group M) (*n* = 43). Randomized group allocation was performed using a computerized randomization table created by one staff member who was not involved in the patients’ anesthesia or recovery care. The randomization result was kept sealed in an envelope; only the nurse who prepared the anesthetics could open the envelope in order to prepare the allocated drug. A total of 83 medical records were analyzed, 43 from group D and 40 from group M. The patients, the nurse in the post-anesthesia care unit (PACU), and attending anesthesiologists were blinded to the medicine administration.

### Inclusion criteria

We enrolled 86 adult male patients with American Society of Anesthesiologist physical status III or IV, aged 25–70 years, scheduled for partial or total laryngectomy.

### Exclusion criteria

Patients with cardiac disease, neuropsychiatric diseases, pharyngeal paraganglioma, or uncontrolled hypertension (i.e., systolic blood pressure > 160 mmHg or diastolic blood pressure > 90 mmHg), taking β-adrenoreceptor blockers, with long-term (> 6 months) abuse of alcohol, taking opioids or sedative-hypnotic drugs, with dexmedetomidine or midazolam allergies, undergoing awake fiberoptic intubation, with operation times shorter than 1 h or longer than 4 h, or with a tracheotomy history were excluded.

### Anesthesia

Drugs, including sedative, analgesic, anti-emetic, and anti-itching drugs, were not given before an operation. After arrival at the operation room, the electrocardiogram, SpO_2_ levels, blood pressure, the bispectral index, end-tidal carbon dioxide levels, and temperature were continuously monitored and recorded. General anesthesia was induced with sufentanil (0.2 μg/kg) and propofol (2.5 mg/kg), and after confirmation of adequate muscle relaxation with the administration of cisatracurium (0.2 mg/kg) iv, an endotracheal tube with an internal diameter of 7 mm was inserted into the trachea. Endotracheal tube cuff pressure was maintained at 25 cmH_2_O measured using a calibrated handheld Portex Cuff Inflator Pressure Gauge (Portex Limited, Hythe, Kent, UK). Prior to the start of surgery, sufentanil (0.1 μg/kg) was given. Either dexmedetomidine (Precedex; Henrui Pharmaceutical, China) (group D, *n* = 43) infusion (0.5 μg/kg 10 min before tracheotomy, then adjusted to 0.3 μg/kg/h) or midazolam (Midazuolun injection; Enhua Pharmaceutical, China) (group M, *n* = 43) infusion (0.05 mg/kg 10 min before tracheotomy, then adjusted to 0.02 mg/kg^/^h) was administered in a blind mode. Anesthesia was maintained with a minimum alveolar end-tidal concentration of sevoflurane of 1–1.3 in 30% oxygen/air mixture to keep the bispectral index between 45 and 55. The maintenance infusion rate of cisatracurium was 1–1.5 μg/kg/min and the maintenance infusion rate of sufentanil was 0.002 μg/kg/min according to clinical needs. Granisetron (6 mg) was administered at the end of surgery to prevent post-operative nausea and vomiting (PONV). Endotracheal secretions were removed before tracheostomy tube insertion. Topical tetracaine hydrochloride gel was applied to the tracheostomy tube to enhance toleration.

After surgical procedures were finished, sevoflurane administration was discontinued, 100% oxygen was administered at 6 l/min, and patients were transferred to the PACU. Neostigmine (0.04 mg/kg) and atropine (0.02 mg/kg) were given to reverse residual neuromuscular block. After spontaneous ventilation returned, patients were considered to have fully recovered from muscle relaxation, and after patients opened their eyes, they were weaned from mechanical ventilation. Nurses who assessed subjects were blinded to the medicine intervention. If there was any adverse event, an attending anesthesiologist managed it. In the case of bradycardia (heart rate [HR] < 45 beats/min), 0.5 mg atropine was administered, and if systolic blood pressure (SBP) decreased to less than 90 mmHg, ephedrine (6 mg) was used.

Cough grading was based on a modified 4-point Minogue scale: grade 1, no cough; grade 2 (mild), coughing once or twice; grade 3 (moderate), fewer than 4 non-sustained coughs lasting 1–2 s each or overall coughing lasting less than 5 s; grade 4 (severe), at least 4 coughs lasting at least 2 s, or overall coughing duration more than 5 s [8]. Patients with grades 3 and 4 were categorized as “moderate to severe.” The patients’ levels of sedation were assessed by the Ramsay sedation scale (RSS): 1, the patient is anxious and restless or agitated, or both; 2, the patient is cooperative, tranquil, and oriented; 3, the patient responds to commands only; 4, the patient exhibits a brisk response to loud auditory stimuli or a light glabellar tap; 5, the patient exhibits a sluggish response to a loud auditory stimulus or a light glabellar tap; 6, the patient exhibits no response [9]. In addition, the nurse also assessed the post-operative pain score by a visual analog scale (VAS) (on a scale from 0 to 10, where 0 is no pain, and 10 is very much pain). If the pain score was above 5, sufentanil (0.1 μg/kg) was given to patients immediately as rescue analgesic; consumption of analgesics was recorded.

HR, SBP, diastolic blood pressure (DBP), and pulse oximetry results (SpO_2_) were recorded before induction (T_1_), after drug administration (T_2_), after intubation (T_3_), after medicine intervention (T_4_), at the moment of laryngectomy (T_5_), after completion of surgery (T_6_), at the point of awareness (T_7_), at departure from the PACU (T_8_), 2 h after surgery (T_9_), 24 h after surgery (T_10_), and 48 h after surgery (T_11_). Duration of surgery, respiratory recovery time, and duration of PACU stay were also recorded. The incidence of adverse events, including bradycardia, hypotension (< 30% decrease from baseline), hypertension (> 30% increase from baseline), vomiting, pale lips, delirium, subcutaneous emphysema, and hematoma, was noted by a nurse who was blinded to medicine intervention. The incidence of pneumonia 72 h after surgery was also recorded.

### Statistical analysis

The primary endpoint was incidence and severity of cough. The secondary outcome measures were hemodynamic responses, post-operative pain scores, sedation scores, respiratory recovery time, duration of PACU stay, and incidence of adverse events.

PASS15 was used to calculate the sample size. On the basis of a preliminary study, the incidence of no cough in group M was about 65%, and in group D it was about 25% higher than in group M. The proportion in group D was assumed to be 0.65 under the null hypothesis and 0.90 under the alternative hypothesis. The proportion in group M was taken as 0.65. The test statistic used is the one-sided Z-test with unpooled variance. The significance level of the test is 0.025. Group sample sizes of 40 in group D and 40 in group M achieve 80% power to detect a difference between the group proportions of 0.25. Assuming a dropout rate of 8%, the final sample size was determined to be 43 patients per group, with a power of 80% and an alpha level of 0.05.

Student’s *t* test was used for between-group comparisons of HR, SBP, DBP, and SpO_2_. Repeated-measures ANOVA was used for within-group comparisons. The χ^2^ test or the Fisher exact test was used to analyze coughing severity, sedation, pain scores, and adverse events. A *P*-value of 0.05 or less was considered statistically significant.

## Results

### Baseline characteristics

#### The incidence and severity of coughing

The prevalence of no coughing was significantly higher in group D than in group M, while patients were at the points of wakefulness (88% [38] vs. 65% [26], *P* = 0.018) and departure (100% [40] vs. 65% [28], *P* = 0.009) (Table 1). No patient in group D and 3 patients in group M experienced severe coughing. The incidence of mild cough was significantly lower in group D than in group M (14% [6] vs. 40% [16] of patients, *P* = 0.015). The incidence of “moderate to severe” cough was significantly higher in group M than in group D (5% [2] vs. 40% [16] of patients, *P* = 0.012).

**Table 1:** The incidence and severity of coughing in PACU

	No Cough		Mild Cough		Moderate Cough		Severe Cough	
	Awake	Departure	Awake	Departure	Awake	Departure	Awake	Departure
Group D	38*	40**	3	3	2^&^	0	0	0
Group M	26	28	7	9	5	2	2	1

*p = 0.018 vs Group M (awake)

**p = 0.009 vs Group M (departure)

Moderate+Severe, ^&^*p* = 0.012 vs Group M

#### Perioperative hemodynamic changes

**Fig. 2:**
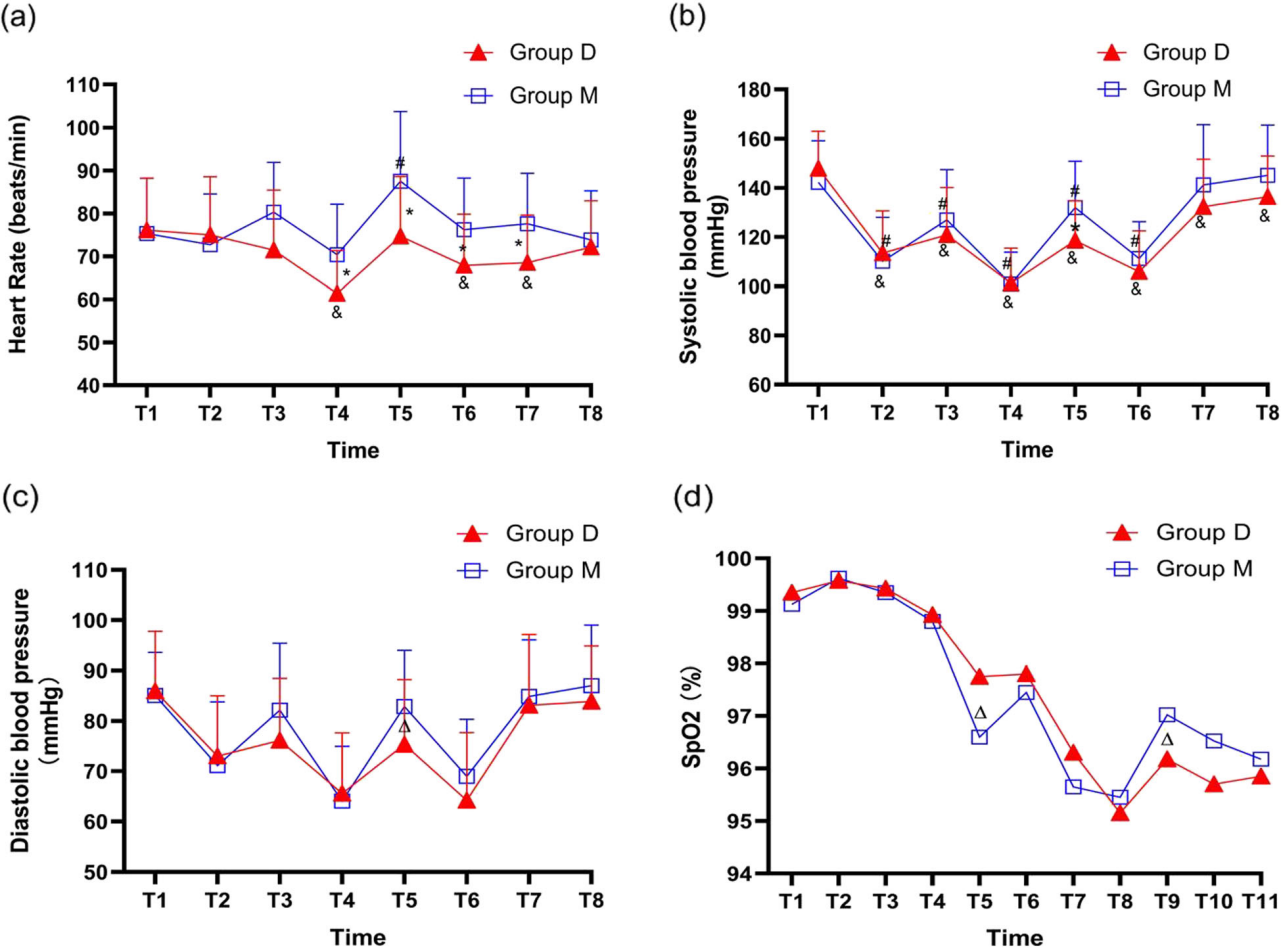
Hemodynamic changes. **a** Comparison of mean HR. **b** Comparison of mean SBP. **c** Comparison of mean DBP. **d** Comparison of mean SpO_2_. *P < 0.01; ^&^*P* < 0.01 group D versus T_1_; ^#^*P* < 0.01 group M versus T_1_; ^Δ^*P* < 0.05

In group M, HR at T_3_, T_4_, T_5_, T_6_, and T_7_ was significantly higher/lower than in group D. HR in group M was significantly higher at the moment of intubation and at the moment of laryngectomy compared with values before anesthesia (Fig. 2a).

As shown in Fig. 2b and c, SBP (131.98 vs. 120.33, *P* = 0.005) and DBP (82.88 vs. 76.98, *P* = 0.042) were significantly higher in group M than in group D at the moment of laryngectomy. Compared with pre-anesthesia values, SBP was significantly lower at other moments (*P* < 0.01) in group D. In group M, compared with pre-induction values, SBP was significantly lower at T_2_, T_3_, T_4_, T_5_, and T_6_ (*P* < 0.01), but was not significantly different at the points of wakefulness and departure from the PACU (*P* > 0.05).

SpO_2_ was significantly higher in group D than in group M at the moment of laryngectomy (97.77 vs. 96.60, *P* = 0.040). However, at 2 h post-surgery, SpO_2_ was significantly higher in group M than in group D (96.14 vs. 97.03, *P* = 0.041) (Fig. 2d). Desaturation (SpO_2_ < 92%) was observed in 5 patients in group M and no patient in group D at the point of laryngectomy (*P* = 0.029) (Supplemental Table 2). The overall incidence of desaturation was lower in group D than in group M (20 [47%] vs. 29 [72%], *P* = 0.029) (Supplemental Table 2).

There was no significant difference in time to respiratory recovery and duration of PACU stay between the two groups (Table 2). As shown in Table 3, RSS at wakefulness in the PACU was higher in group D than in group M (1.98 vs. 1.80, *P* = 0.025).

**Table 2:** Recovery Profiles

Time	Group D(*n* = 43)	Group M(*n* = 40)	P
Recovery time (min)	26.6 ± 12.1	28.5 ± 12.3	0.475
Awake time (min)	46.5 ± 16.0	46.2 ± 14.7	0.938

Values are mean ± SD or number

**Table 3:** Ramsay score

Ramsay score(1/2/3)	Group D(*n* = 43)	Group M(*n* = 40)	P
Awake	1/42/0	8/32/0	0.025
Departure	1/42/0	1/39/0	1.00
2 h after surgery	0/43/0	1/38/1	0.332

Grade of sedation, 1 = anxious and restless or agitated, 2 = cooperative, tranquil, and oriented, 3 = responds to commands only

### Post-operative pain score

We observed no significant difference in post-operative pain scores between the two groups at the point of wakefulness. The requirement for rescue analgesics was significantly lower in group D than in group M (4 vs. 13, *P* = 0.013) (Table 4). But the post-operative pain score was significantly lower in group D than in group M at the moment of departure from the PACU (*1.2* vs *2.0, p < 0.05*), the difference is statistically significant but not clinically significant, patient who suffer postoperative pain in the PACU has been given rescue analgesics to relieve the pain, the number of patients who has been given rescue analgesics was significantly higher in group M than in group D (*13* vs *4, p < 0.05*).

**Table 4:** Pain scores and postoperative requirement for rescue analgesics at PACU

	Group D(*n* = 43)	Group M(*n* = 40)	P
VAS score (T_7_)	1.6 ± 1.6	2.0 ± 2.0	0.261
VAS score (T_8_)	1.2 ± 0.9	2.0 ± 2.0	0.024
Rescue analgesics(n)	4	13	0.013

Values are mean ± SD or number

### Adverse events

The incidence of postoperative complications are presented in supplemental Table 3. There was no significant difference between two groups. Vomiting was noted in 7 patients in group D and 12 in group M. Hypertension was observed in 1 patient in each group. Pale lips was observed in 2 patients in group D, severe bradycardia in 1 patient in group D, delirium was reported in 2 patients in group M, subcutaneous emphysema in 1 patient in group M, while re-exploration of operation site for hematoma was observed in 1 patient in group M. Postoperative pneumonia was noted in 1 patient in group M. During the operation, vasopressor was used in 18 patients in group D and 11 in group M, there was no significant difference in between two groups (*18* vs *11*, *p = 0.249*).

## Discussion

This study showed dexmedetomidine provided adequate and satisfactory coughing suppression, stable hemodynamics, and good recovery for patients undergoing partial and total laryngectomy.

Coughing after laryngectomy is always related to airway secretions or the presence of a tracheostomy tube; we therefore used suction to remove oral and airway secretions before tube insertion. We used topical tetracaine hydrochloride gel for the tracheostomy tube to reduce tube stimulation of the peripheral nervous system. The prevalence of no coughing was significantly higher in group D than in group M. No patient in group D experienced severe coughing. This result is consistent with a previous study, which showed that dexmedetomidine is effective in attenuating the airway reflex to tracheal extubation [10]. Several pharmacological agents have been reported to decrease coughing, including lidocaine and opioids. Recently, a systematic review has been sponsored/carried out to investigate optimal pharmacological methods for reducing coughing after general anesthesia [11]. Opioids such as remifentanil and fentanyl are commonly used to prevent cough, but opioids can produce undesirable adverse events, such as respiratory depression, delayed awakening, and PONV. In the present study, we observed no significant differences in time to respiratory recovery and duration of PACU stay between the two groups, but the post-operative pain score was significantly lower in group D than in group M at the moment of departure from the PACU, fewer patients were given rescue analgesics to relieve the pain, and dexmedetomidine did not appear to induce respiratory depression.

Blunting the cardiovascular response can decrease the incidence of complications. Compared with group M, there was a significant decrease in HR, SBP, and DBP at the moment of laryngectomy. We did not combine dexmedetomidine and remifentanil due to the possibility of delayed awakening [12]. Dexmedetomidine can reduce the release of norepinephrine, resulting in decreased catecholamine release from nerve endings and a resultant central sympatholytic effect, leading to decreases in HR and blood pressure [13]. However, dexmedetomidine also has some disadvantages, including inducing bradycardia and hypotension in old patients. Severe bradycardia was observed in 1 patient in group D. A vasopressor was used in 18 patients in group D and 11 patients in group M. Hypotension and bradycardia occurred more often with the initial dose in group D. Previous studies have reported that midazolam had no significant effects on sympathetic tone but slightly decreased blood pressure for about 10 min due to decreased systemic vascular resistance and myocardial contractility [14, 15]. We speculated that a lower dose of dexmedetomidine may be more appropriate to elderly patients. SpO_2_ levels were significantly higher in group D than in group M at the moment of laryngectomy. This may be attributed to decreased HR-induced lower oxygen consumption. Animal experiments have shown that dexmedetomidine pre-conditioning exerts cardioprotective effects against hypoxia injury and can improve peri-operative hypoxemia [16, 17].

Emergence agitation can result in cardiovascular instability, decreased venous return and increased intracranial pressure, decreased functional residual capacity, wound dehiscence, and hemorrhage [18]. Midazolam is an effective sedative anxiolytic that provides anterograde amnesia. But it has been reported that there is a high risk of drug accumulation and delirium when using midazolam in patients with liver dysfunction [19]. In our research, delirium was observed in 2 patients in group M, but this was relieved within 24 h. We found satisfactory sedation with dexmedetomidine. Dexmedetomidine exhibits a high specificity for α_2_ vs. α_1_ receptors [20], producing unique sedative effects similar to normal sleep. Dexmedetomidine has been reported to be used for long-term sedation during mechanical ventilation in critically ill patients at the intensive care unit and for decreasing patient agitation in the PACU [21]. Our results also confirmed that dexmedetomidine may be an effective agent for sedation in partial and total laryngectomy.

Partial and total laryngectomy is associated with a high level of pain [22]. Our results show that the post-operative pain scores and the requirement for rescue analgesics were significantly lower in group D than in group M. However, the analgesic efficacy of dexmedetomidine is still controversial [23], and the analgesic mechanism of dexmedetomidine remains to be further studied.

Although there were more adverse events in group M compared with group D, this difference was not statistically significant. Insertion of a stomach tube is also a risk factor of PONV, but all patients retained the stomach tube in our study. Patients who breathe via a tracheostomy tube cannot make use of their glottis [24]. Preservation of the cough reflex is mandatory to prevent pulmonary complications [25]. No patient developed pneumonia in group D. The finding that the prevalence of post-operative pneumonia did not differ between the two groups suggests that dexmedetomidine is not associated with post-operative pulmonary infections.

There were several limitations to the present study. The major limitation was that we investigated only one dose of dexmedetomidine. Second, the sample size was relatively small, so future multicenter studies comprising larger sample sizes are needed. Third, we did not include patients with bilateral cervical lymph node dissection, since the wounds are always larger, so our results are not generalizable to these patients. Fourth, we did not include patients older than 70, while in Europe and North America, approximately 30% of all head and neck cancer patients are aged over 70 years [26]. Finally, there were potential sources of heterogeneity, including the fitness of the patient’s trachea and tracheostomy tube and the fact that surgery was performed by different surgeons.

## Conclusions

In conclusion, intra-operative infusion of dexmedetomidine has advantages, including blunting the airway reflex, good sedation, stable hemodynamics, and a low risk of adverse events. Dexmedetomidine improved the outcome, alleviated patient discomfort caused by the tracheostomy tube, and allowed for a smooth emergence from anesthesia.

## Supplementary information

**Additional file 1: Table S1.** Demographics and baseline variables of patients in the two groups.

**Additional file 2: Table S2.** Oxygen desaturation (SpO2 < 92%).

**Additional file 3: Table S3.** Incidence of drug-related adverse events.

## Data Availability

The datasets used and/or analysed during the current study are available from the corresponding author on reasonable request.

## Abbreviations

group D: Group dexmedetomidine
group M: Group midazolam
PACU: Postanesthesia care unit
SpO_2_: Pulse oximetry
HR: Heart rate
SBP: Systolic blood pressure
DBP: Diastolic blood pressure
VAS: Visual Analogue Scale
RSS: Ramsay sedation scale
ASA: American Society of Anesthesiologist physical status
PONV: Postoperative nausea and vomiting
