# Supplementary material for: Dexmedetomidine versus midazolam on cough and recovery quality after partial and total laryngectomy – a randomized controlled trial
Source: BMC Anesthesiol. 2020 Sep 28;20:249. doi: 10.1186/s12871-020-01168-7 (PMC7523379; doi:10.1186/s12871-020-01168-7)
Supplement: Supplementary file 1 — Additional file 1: Table S1. Demographics and baseline variables of patients in the two groups. [file 12871_2020_1168_MOESM1_ESM.docx]

**Supplemental table 1. Demographics and baseline variables of patients in the two groups**

| Variable Group D(n=43) Group M(n=40) P |
| --- |
| **Age (years)** 58.9±8.4 61.3±11.0 0.255  **Sex male(%)** 43(100%) 40(100%) ---  **Weight (kg)** 67.3±9.1 67.6±7.9 0.865  **Height(cm)** 170.3±5.7 169.1±4.7 0.338  **Duration of surgery (min)** 138.1±53.5 135.4±50.1 0.809  **Surgery type performed** 0.273  Partial laryngectomy 24 18  Partial laryngectomy+ neck dissection 4 8  Total laryngectomy 5 7  Total laryngectomy+ neck dissection 6 11  **Smoking history**  43 40 ---  **Hypertension** 17 12 0.363  **Diabetes mellitus** 3 4 0.620 |

Values are mean±SD or numbers.
