# Supplementary material for: Dexmedetomidine versus midazolam on cough and recovery quality after partial and total laryngectomy – a randomized controlled trial
Source: BMC Anesthesiol. 2020 Sep 28;20:249. doi: 10.1186/s12871-020-01168-7 (PMC7523379; doi:10.1186/s12871-020-01168-7)
Supplement: Supplementary file 2 — Additional file 2: Table S2. Oxygen desaturation (SpO2 < 92%). [file 12871_2020_1168_MOESM2_ESM.docx]

**Supplemental table 2.Oxygen desaturation (SpO_2_ <92%)**

|  | T_5_ | T_6_ | T_7_ | T_8_ | T_9_ | T_10_ | T_11_ | Total |
| --- | --- | --- | --- | --- | --- | --- | --- | --- |
| GroupD | 0 | 3 | 4 | 6 | 2 | 3 | 2 | 20(43) |
| GroupM | 5 | 3 | 9 | 6 | 0 | 2 | 4 | 29(40) |
| P value | 0.023 | -- | -- | -- | -- | -- | -- | 0.029 |

--:p>0.05.
