# Supplementary material for: Dexmedetomidine versus midazolam on cough and recovery quality after partial and total laryngectomy – a randomized controlled trial
Source: BMC Anesthesiol. 2020 Sep 28;20:249. doi: 10.1186/s12871-020-01168-7 (PMC7523379; doi:10.1186/s12871-020-01168-7)
Supplement: Supplementary file 3 — Additional file 3: Table S3. Incidence of drug-related adverse events. [file 12871_2020_1168_MOESM3_ESM.docx]

**Supplemental table 3. Incidence of drug-related adverse events**

|  | Group D(n=43) | Group M(n=40) | P |
| --- | --- | --- | --- |
| vomiting | 7（16.3） | 12（30.0） | 0.192 |
| Hypertension | 1（2.3） | 1（2.5） | 1.00 |
| Severe bradycardia | 1（2.3） | 0（0） | 1.00 |
| Others | 2（4.7） | 3（7.5） | 0.668 |
